# Evaluating Potential Biomarkers of Health and Performance in Veal Calves

**DOI:** 10.3389/fvets.2018.00133

**Published:** 2018-06-21

**Authors:** Francesca Marcato, Henry van den Brand, Bas Kemp, Kees van Reenen

**Affiliations:** ^1^Adaptation Physiology Group, Wageningen University & Research, Wageningen, Netherlands; ^2^Animal Production Systems Group, Livestock Research, Wageningen University & Research, Wageningen, Netherlands

**Keywords:** veal calves, challenges, health, diseases, biomarkers, stress

## Abstract

Veal calves undergo many challenges in the early stages of their life. Such challenges, including mixing procedures and transportation of calves to the veal farm, may have a negative influence on growth rate, feed intake, metabolism, immunity and disease susceptibility of calves. As a consequence, many hematological, physiological, metabolic and immunological parameters of stressed calves might be altered on arrival at the veal farm. Some of these response variables might be useful as biomarkers of performance of calves at the veal farm as they might provide information about an ongoing disease process, or may predict future diseases. Biomarkers might be helpful to group and manage calves in different risk categories after arrival. By adopting treatment decisions and protocols on a risk-group or individual basis, it would be possible to improve animal health and reduce both disease incidence and antibiotic use. Moreover, the use of biomarkers might be an economically feasible approach as some of them do not need invasive techniques and others can be measured in blood already taken during routine checks. Previous literature mainly assessed the physiological responses of calves to transportation. However, information on the link between on-farm arrival data and future health and performance of veal calves is limited. This review, therefore, examined a wide range of papers and aimed to identify potential biomarkers of future health and performance.

## Introduction

The veal industry plays an important role worldwide as side market of the dairy industry (1). Europe is the main veal producer, accounting for 82% of the global production in 2010. Within the European context France, the Netherlands and Italy are the leading veal producing countries with a global market share of 27, 25, and 16%, respectively (2). Belgium and Germany represent 6 and 5% of global veal production, whereas other European countries either have a small veal sector such as Switzerland, or no veal production due to animal welfare restrictions such as the Scandinavian countries. Outside Europe, veal production is relatively limited; main veal producing countries outside Europe include the United States, with Canada, Australia and New Zealand each accounting for 3 to 6% of global production (1). The current review will focus on the European scenario because Europe is the leader in veal production.

White veal calves face many challenges in the pre-weaning period (3). These challenges include birth, transportation, mixing procedures, inappropriate management conditions and new housing environments (4). At the dairy farm, separation of calves from their dams usually occurs immediately after birth. Subsequently, when calves are 14–20 days of age, they are gathered from different dairy farms and transported to a collection center, followed by another transport to the veal farm (4). During these phases, calves from different farms are mixed and are exposed to new environmental conditions and management practices. All these challenges occur at an age at which the calf is immature and several physiological systems are still developing. For example, young calves are still developing their gastrointestinal tract (GIT), and their thermoregulatory (5) and acquired immune systems (6) are not completely functional yet. During the first week of life calves may be exposed to pathogens against which they may not have (maternal) antibodies (7, 8). The combination of the indicated challenges and the immature physiological systems of the calves may explain the high susceptibility of calves to infections. As a result, calf health and performance at the veal farm are affected (3, 9). Calves may develop diseases, among which respiratory diseases (e.g., bovine respiratory diseases, BRD) and enteric diseases are most frequently observed (10–12).

Respiratory diseases are common health disorders in veal calves, which have a severe impact on both animal welfare and the income of producers, because they are the most important causes of morbidity and mortality (13, 14). According to Pardon et al. (11), BRD incidence in veal calves during the rearing period ranges between 4.6 and 43.8%, with an average of 17%. The same authors reported that of the 5.7% of veal calves which died before the end of the production cycle, 27.1% had suffered of pneumonia. Approximately two thirds of calves diagnosed with pneumonia were individually treated for BRD. Post-mortem analysis of lungs at slaughter (4) revealed that 21.4% of veal calves showed signs of pleuritis and 13.9 and 7.7% signs of pneumonia, respectively. Bovine respiratory disease is characterized by many clinical signs, including nasal discharge, coughing, fever, inappetence, apathy and hampered respiration (4). Both subclinical and clinical signs hamper the growth and welfare of infected calves compared with healthy animals (15). Bovine respiratory disease is a complex disease that depends on different interacting factors. The etiology of this respiratory disorder involves several infectious agents, such as bacteria, mycoplasma and viruses, that act in synergy with stressors, like weaning, transportation, nutrition and rearing environment (16, 17). Viruses which contribute to the outspread of BRD are mainly bovine respiratory syncytial virus (BRSV), para-influenza-3 virus (PI3V) and bovine viral diarrhea virus (BVDV) (16).

Enteritis is another disease that is frequently diagnosed during early stages of life and it is particularly seen in the first 3 weeks after arrival of calves at the veal farm (11). Pardon et al. (11) showed that, of 5.7% of calves which died, 7.5% had suffered from enteritis. Different microorganisms, including bacteria, viruses, protozoa and yeasts are responsible for enteric diseases (18). *Escherichia coli, Salmonella* sp., and rotavirus are the most common microorganisms, causing enteric diseases and diarrhea in young calves (19).

In an attempt to counteract the negative effects of diseases, the use of therapeutic treatments has become widespread (20). The use of antimicrobial growth promoters has been banned in Europe since 2006 (21), but since then the use of therapeutic antimicrobials increased (22). A recent study demonstrated that antimicrobial use in veal calves is the highest of all food producing animals (10). Pardon et al. (23) reported that in Belgium the antimicrobial consumption in white veal calves is approximately 25.2 tons per year. In the Netherlands, one of the main veal producing countries in Europe, that has similar veal production systems as Belgium, a reduction in antimicrobial use in veal production has already been achieved during recent years. However, the usage of antimicrobials is considered still high (10, 24). There is growing public concern about the consequences of feeding antibiotics (especially oral treatments) to farm animals, including veal calves, for both human and animal health (e.g., a massive use of antibiotics may cause antibiotic resistance) (20, 25). Therefore, there is a strong need for management strategies in the veal sector that may help to reduce the incidence of diseases and, consequently, antibiotic use.

## Clinical utility of potential biomarkers in veal calves: future implications

This review builds on the idea that response variables obtained in calves on arrival at the veal farm may be used as predictors or biomarkers of later health and performance. A biomarker, per definition, is a marker of a biological process or state and it can provide information on a current status or future risk of disease of an individual (26). The availability of such biomarkers would be helpful, for example, to identify individual calves at an early stage with an enhanced probability to develop disease, and to take preventive measures before clinical problems occur. At herd level, biomarkers might be used for profiling calves according to the magnitude of stress they have experienced and their predisposition to develop future diseases (27). Grouping of calves in different risk categories should help the farmer in managing calves at arrival. By adopting handling procedures, treatment decisions and protocols on a risk-group or individual basis, farmers might be able to better meet individual animal needs and improve the health and welfare of calves throughout the veal production chain [see also Renaud et al. 28). Collectively, this may reduce the incidence of disease as well as the use of antibiotics.

Previous studies (29–32) examined effects of different transport conditions and duration on calf blood constituents and performance of calves at their arrival at the veal farm. Only a limited number of studies (30, 33, 34) assessed relationships between on-arrival blood constituents and future performance of calves at the veal farm. By examining a much wider range of papers, this review aimed to identify potential on-farm biomarkers of health and performance of calves at the veal farm.

## Effects of environmental challenges on physiological pathways and on biomarkers

**Figure 1:**
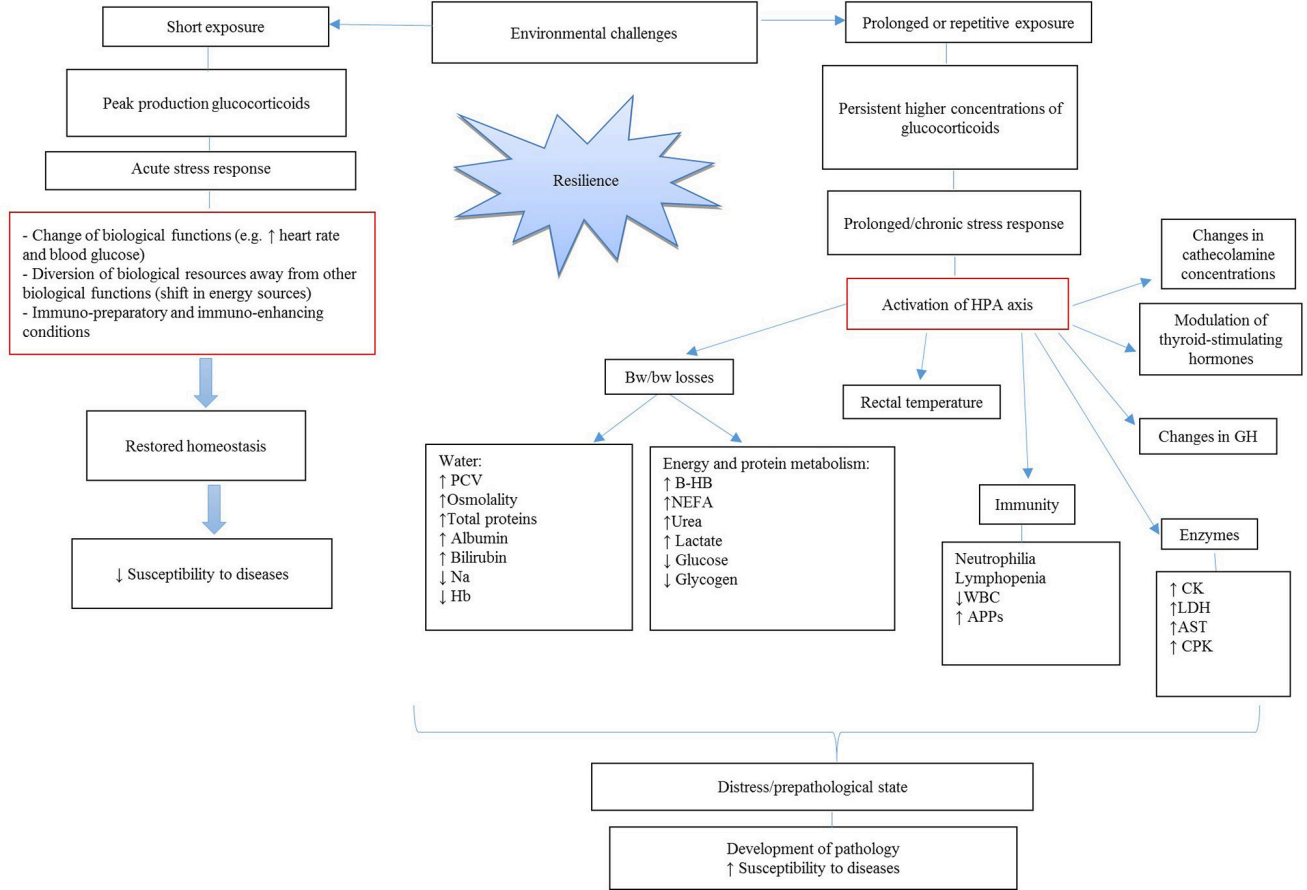
Effects of short and long-term exposure to environmental challenges on disease susceptibility. HPA axis, hypothalamic-pituitary-adrenal axis; GH, growth hormone; BW, body weight; PCV, packed cell volume; Hb, hemoglobin; β-HB, β- hydroxybutyrate; NEFA, non-esterified fatty acids; WBC, white blood cells; APPs, acute phase proteins; CK, creatine kinase; LDH, lactate dehydrogenase; AST, aspartate aminotransferase; CPK, creatine phosphokinase.

Environmental challenges, including road transportation, are known to affect metabolic (35), physiological (36), immunological (37–39) and behavioral responses (36, 40) of calves. As illustrated in Figure 1, exposure of the animal to environmental challenges can be short-term or prolonged. In both cases, an increase in plasma concentrations of glucocorticoids and cortisol is observed. In case of short exposure, a peak production in glucocorticoids determines an acute stress response. As a result, a calf might experience changes in its biological functions, with shifts in energy sources that allow the animal to better cope with the stressor. Moreover, an activation of the immune system, including enhanced cell function, cell-mediated, humoral and innate immunity, might protect calf health (41). All these changes might restore homeostasis in the short-term and not affect animal health and welfare on the long run. In case of prolonged exposure, persistent higher concentrations of glucocorticoids may lead to prolonged/chronic stress response (Figure 1). Under these circumstances, the activation of the hypothalamic-pituitary-adrenal (HPA) axis is responsible for long-lasting effects on the animal body. Effects include changes in catecholamine release, growth hormone (GH) secretion and modulation of thyroid-stimulating hormones. Additionally, calves might experience BW losses due to increased dehydration and nutrient mobilization accompanied by changes in rectal temperature, enzymes concentrations in the blood and a suppression of the immune function. As a consequence of prolonged stress exposure, calves may experience changes of biological functions to an extent that the risk of developing diseases is increased (42). This review will first focus on the main effects of HPA axis activation as one of the main pathways between exposure to environmental challenges and susceptibility to disease will be discussed. Therefore, a description in changes in cortisol and BW will be reported. The current review will first discuss dehydration-related variables and then variables associated with nutrient metabolism. Then changes in rectal temperature, immune cells and enzyme concentrations will be discussed. Interactions between different physiological variables and details about the corresponding biological mechanisms involved will be also explained. All these effects will be discussed in association with disease development in young veal calves in the first 3 weeks at the veal farm. Moreover, it will be discussed which parameters might be the most important biomarkers that could be used at on-farm arrival to predict health and welfare of calves at the veal farm. For achieving these goals, each paragraph of this review will contain a description of the physiological role of the variable of interest, how it is affected by environmental challenges, and the possible association of the variable with an ongoing disease process or later disease susceptibility. Finally, some conclusive remarks on the potential use of the variables as biomarker will be made and some advices for future studies will be given.

## Cortisol

Cortisol is a glucocorticoid hormone derived from cholesterol. Cortisol is the primary hormone involved in the stress response and is regulated by the HPA axis. The main action of cortisol consists of activating biological functions to respond to stress and restoring homeostasis after exposure to stress (44). Transportation of calves to the veal farm, as well as management of calves at the dairy farm of origin and at the collection center, are challenges that increase the activity of the HPA axis (36). A rise in plasma cortisol concentrations was often observed in transported calves and it is the main indicator of psychological/physiological stress (Figure 1) (45, 46). The increase in plasma cortisol concentrations can be transient when normal levels are restored in 1–2 days or chronic when hyper-cortisolemia continues for at least 4–5 days (47). Grigor et al. (31) found significantly higher concentrations of cortisol in transported 10-days-old calves (up to 25.2 nmol/l) compared with non-transported calves (up to 16.3 nmol/l). Bernardini et al. (48) also observed an increase in cortisol (up to 23.7 nM) in young calves (37 ± 6 days of age) following transportation for 19 h. However, cortisol concentrations were not extremely high compared to normal levels (18.4 nM) and calves restored their basal cortisol levels within 2 days.

Aich et al. (49) found considerably higher cortisol levels the day prior to BRD infection in animals that died compared to those surviving a synergic viral-bacterial infection. However, these concentrations (150 mmol/l cortisol in serum) are indicative of the current health status of the animal and not maintained beyond that day. In all the previously mentioned studies the rise in cortisol concentrations was observed on a short-term and there was no information on chronic elevations of cortisol.

**Figure 2:**
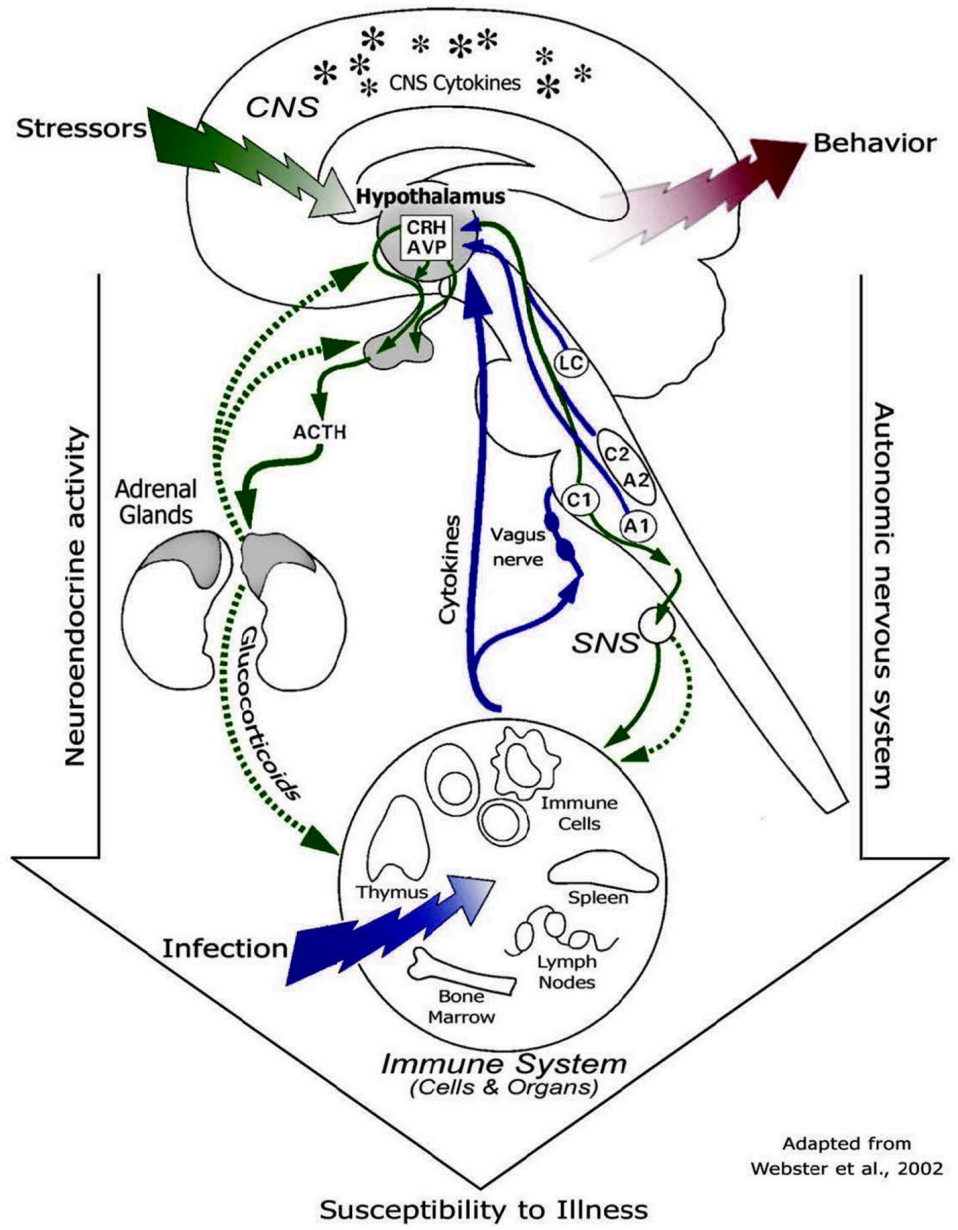
Diagram of routes of communication between the brain and the immune system, including HPA axis, sympathetic nervous system, and cytokine feedback to the brain [© 2011. Oscar Vegas, Larraitz Garmendia, Amaia Arregi and Arantza Azpiroz. Originally published in “Effects of social stress on immunomodulation and tumor development” under CC BY 3.0 license. Available from Vegas et al. (43)].

Different studies (47, 50) reported that prolonged high cortisol concentrations can result in increased glucose metabolism, insulin resistance, inhibition of glycogen synthase in the skeletal muscle and visceral obesity. As a consequence of these adverse health effects, the animal might be less resilient to diseases. These metabolic changes might lead to problems, including hyperglycemia, insulin resistance, glucosuria and reduced energy utilization at the end of the producing cycle at the veal farm. Moreover, changes in circulating glucocorticoids concentrations, and thus cortisol, are responsible for changes in cytokine levels and the production by leukocytes (51–53). Therefore, when calves are stressed and have high cortisol levels for a prolonged period, calves are at risk for an altered immune function (43). Figure 2 shows the pathways through which glucocorticoids affect the immune system and, thus susceptibility to diseases (43).

Cortisol, by impairing the immune functionality, might be used as biomarker for predicting future diseases. Calves with chronic hyper-cortisolemia and changes in their immune cell parameters (e.g., neutrophils, lymphocytes and acute phase proteins, APPs) might be profiled as high-risk calves. Further research is needed to establish a relationship between chronic high cortisol concentrations upon arrival at the veal farm and incidences of health and metabolic disorders during the subsequent fattening period (4–5 months). Chronic hyper-cortisolemia might be measured by taking repetitive blood samples, for example, in the first 2 weeks after arrival at the veal farm. Then, it should be checked whether the overall cortisol levels in serum are consistently increased during the period of blood collection. Moreover, future studies should address the relationship between chronic hyper-cortisolemia and functionality of the immune system. There is a need to clarify what type of immune cells have the greatest effects on the health of veal calves, and especially on the probability of developing respiratory or enteric diseases. All this information will provide useful data for clarifying the role of cortisol as biomarker of future diseases. However, chronic hyper-cortisolemia can only be assessed by repeated measurements. With regards to feasibility, it would be preferable to use non-invasive techniques such as collecting saliva (54) or hair (55) samples instead of blood to prevent induction of acute stress response during sampling.

## Body weight (BW) and BW losses

Measurement of BW losses, defined as the differences in BW before and after transport, is an indicator of the hydration status and/or body nutrient mobilization (e.g., fat or proteins). Environmental challenges, and especially long transport durations, are important causes of dehydration, fat mobilization, muscle protein degradation and thus loss in BW (Figure 1) (36). Therefore, BW before transport and upon arrival at the veal farm may reflect the dehydration and metabolic state of the animal. Body weight *per se*, is related to the condition and birth weight of the animal. Calf BW on arrival at the farm might also influence the performance of the animal in the first weeks of the producing cycle.

It has been reported that calves may lose between 3 and 11% of their BW during transport to the farm (56). Bernardini et al. (48) found that calves unloaded at the veal farm after 19 h transport had 6.4% BW losses compared with non-transported calves. Calves may lose BW after long transport durations because of stress coupled with fasting and mild dehydration (57, 58). Calves that lose more than 8% of BW in 1 day are depressed (clinical symptoms: skin tenting >10 s, eyes very sunken, dry gums and the calf lays down) and require intravenous treatment; BW losses >14% might even lead to calf death (59). There is evidence that BW loss is highly influenced by feeding and water provision prior to transport of calves to the veal farm (30, 48). Moreover, an increase in plasma protein, albumin, osmolality and packed cell volume (PCV) in calves with high BW loss, suggests that BW losses are particularly due to dehydration of calves.

Body weight losses, resulting from dehydration might reduce the adaptive capacity of the animal. According to Renaud et al. (28) the risk of mortality increases when calves are >10% dehydrated. However, mechanisms underlying the loss of BW as a reflection of dehydration have to be further explored.

It was reported that BW at arrival is associated with the prevalence of respiratory diseases at 3 weeks upon arrival at the farm (4). As shown in Table 1, veal calves with *BW* > 51 kg had the lowest probability to develop respiratory diseases at the veal farm (adjusted *R*^2^ = 25%) (4). In recent Canadian studies (28, 60, 61) body weight of calves at arrival at the veal farm was also inversely associated with early mortality (Table 1). Other studies on feedlot cattle used mean cohort arrival body weight as a useful predictor of future diseases and health problems (62–64). Lighter-weight cattle presented a higher incidence of BRD morbidity and overall mortality compared to heavier cattle (62) (Table 1).

**Table 1:** Some associations between body weight (BW) and future risk of respiratory diseases or early mortality in different studies on cattle.

**References**	**Mean arrival BW (kg)**	**Prevalence (predicted means) of respiratory diseases (%)**		***P*-value**
(4)	≤43 43–47 48–51 >51	7.6 6.1 6.6 2.7		0.004
	**BW at arrival**	**Hazard ratio for early mortality (**<**21 days after arrival at the veal farm)**		
(60)	Per 1-kg increase	0.93		< 0.01
	**BW at arrival**	**Odds ratio for early mortality (**<**21 days after arrival at the veal farm)**		
(61)	Per pound	0.99		0.03
	**Mean arrival BW (kg)**	**Incidence rate ratio (IRR) for BRD^a^ morbidity**	**IRR for mortality**	
(62)	272–317 318–362 >362	1.08 0.69 0.55	0.99 0.71 0.52	<0.05
	**Mean arrival BW (kg)**	**Cumulative mortality risk for BRD**		***P*** >χ^2^
(63)	363–408 318–362 272–317 227–271 182–226 <182	0.02 0.12 −0.01 0.04 0.40 0.44		0.0012

a *Bovine respiratory disease*.

From these findings, it can be concluded that BW might be a useful parameter to monitor and to predict the health status of calves at the veal farm. At this stage, BW upon arrival appears to be the most reliable predictor for future diseases at the veal farm. However, it is still unclear whether lower BW values at arrival are due to lower birth weights of calves or due to substantial BW losses during transportation, or a combination of both. This should be elucidated in future research. As shown in Table 1, it seems that differences in BW in the study of Brscic et al. (4) were likely related to different factors (e.g., BW losses, age, colostrum management), whereas in the other studies on feedlot cattle the difference in BW is mainly caused by a different developmental stage of calves. Breed is another important factor that might be related to differences in BW. For example, there is an increasing demand in the veal industry for Holstein Friesian-beef breed cross breeds. Moreover, future studies should investigate whether BW losses are also predictive of the future health state of veal calves. With regards to feasibility, in case of an equal predictive value of both BW losses and BW, BW values are simpler to obtain than those for BW losses. In fact, for measuring BW, the animal is just weighed one time upon arrival at the veal farm, whereas in case of BW losses it is necessary to record BW before and after transportation of calves to the veal farm. Therefore, BW might be a more practical and feasible biomarker compared with BW losses. On the basis of BW values, a farmer might profile veal calves by separating putatively high-risk calves with a lower BW from the rest of animals. Then, lighter calves might receive a special daily care and management in order to avoid the spread of diseases and a lower production performance compared with healthy calves.

## Dehydration-related variables

### PCV

Part of BW losses or low BW of calves may be due to dehydration. Some specific variables may be related to dehydration, among which packed cell volume (PCV). PCV is a variable related to the number of red blood cells (RBC) in an animal. By definition, PCV is the ratio of the red blood cells to the volume of whole blood (which contains also white blood cells and plasma). PCV can be influenced by environmental challenges, such as transportation of calves to the veal farm. PCV values recorded by Knowles et al. (30) range from 39.8% in control non-transported calves to 40.8% in 24-h transported calves. This finding was confirmed in other studies (36, 65) on long-term transport of young cattle. The increase in PCV can also be found in transported calves in combination with fasting of 48–72 h. This is associated with a greater water loss during transportation as well as the stress of ADAPTING to a new environment (36, 46). By contrast, other studies demonstrated a decrease in PCV in animals transported by road (66, 67). After 8-h transport, calves showed a significantly lower PCV value (33.7%) compared with the control non-transported calves (38.2%) (30). These results might be due to restraint and handling procedures before transport or stressors during transport (68). In fact, an increased cortisol concentration seems to move water from the rumen into the plasma resulting in a decrease in PCV values (66).

Both higher and lower PCV values might be good indicators of an ongoing disease process. Calves with diarrhea might experience excessive fluid losses that lead to higher PCV values (69). By contrast, lower PCV can be used for the diagnosis of anemia or other health problems (70).

As explained previously, PCV values can indicate the extent of dehydration of a calf on arrival at the veal farm. When a calf is dehydrated, it may experience a weight loss. Moreover, if not treated immediately, consequences of dehydration might be still visible in the first weeks at the veal farm. Therefore, the ADG and gain:feed ratio may be negatively affected in the first weeks at the veal farm (71). Seifi et al. (72) demonstrated that calves up to 14 days of age with PCV values at arrival above 44.17% were 4 times more likely to die. This was in agreement with Klee et al. (73) who observed a reduction in treatment efficacy in calves with diarrhea when PCV was above 50%. An accurate analyses of PCV values may, therefore, provide information on calves that need to receive extra care in order to minimize body weight losses and reduction in performance due to water losses in the first weeks at the veal farm. Therefore, by identifying potentially high-risk calves with significantly higher (above 43%) PCV values, PCV might be a reliable biomarker of diseases at the veal farm.

### Total protein (TP)

Alongside with PCV, an increase in total protein (TP) and albumin concentrations in the plasma are also measurements reflecting dehydration of the animal (74). TP is also an important indicator of the amount of colostral proteins in young calves, that is reflecting the immune state of these animals (75). This parameter can be influenced by environmental challenges, including transport duration. Bernardini et al. (48) found significantly higher plasma total protein concentrations (63.9 g/l) in young calves (37 ± 6 d of age) subjected to 19-h transport at their arrival at the veal farm compared to non-transported calves.

Values of TP are important, especially for predicting mortality in the first weeks at the veal farm (76). Naylor et al. (77) observed a significantly lower mortality in the first 5 weeks of age in calves with *TP* > 6.1 g/dl. Moreover, Rea et al. (78) reported that calves with TP < 4.5 g/dl had a higher risk of dying in the first weeks at the farm. By contrast, other studies suggested that TP cannot be considered a reliable indicator of diseases and mortality in calves (75, 79). Hence, TP is difficult to interpret in terms of risks for calf health and performance. Higher levels of TP may indicate higher levels of colostral proteins, which is a positive sign. However, high levels of TP may also be indicative of dehydration, which is a negative sign.

### Albumin

Albumin is the major negative acute phase protein (APP). During the acute phase response, albumin concentrations decrease for the synthesis of positive APP. Hence, albumin is a main source of amino acids that animals can use when necessary and it plays an important role in plasma osmotic pressure (80). As shown by the study of Knowles et al. (34), albumin concentrations increased from 39.8 to 43.1 g/l in calves subjected to transport as a result of dehydration.

Albumin might be used not only as a measure of dehydration, but also as a prognostic marker or to assess the severity of diseases (81–83). For example, low albumin concentrations in dairy cattle were associated with uterine infections (83) and inflammation (84, 85).

Limited research (79) has been done in young veal calves, especially on the predictive value of albumin. The available evidence suggests both high and low albumin values may indicate a risk. Thus, it is necessary to understand to what extent an increase or a decrease in albumin values is associated with diseases or future health problems in veal calves.

### Bilirubin

Bilirubin is a product of heme degradation and it functions as antioxidant (86). This variable is influenced by environmental challenges. Mormede et al. (33) found an increase in plasma bilirubin (5–10 μmol/l) in young calves after being transported to the veal farm; this increase was more pronounced in calves subjected to long-transport duration.

The higher concentrations of this variable might be determined by an increased dehydration of the animals subjected to transport. However, their increase could also be related to a compromised health status of calves. Higher bilirubin concentrations were indicators of impaired hepatic function in dairy cows in a negative energy balance situation and with inflammation (87). Extrapolated to calves, this would imply that calves might experience metabolic changes in the liver as a consequence of stress during transport. However, until now no studies on veal calves examined bilirubin as a possible biomarker of ongoing health problems or predictor of future diseases.

### Electrolytes and minerals

Electrolytes and minerals are responsible for maintaining a good water balance and for normal functioning of essential biochemical processes in the animal body (88). Calves may experience changes in their electrolyte and mineral balance when they are transported under stressful conditions (36). Cattle have a substantial blood buffering ability but during transport they show plasma electrolyte and mineral changes, including sodium, potassium, chloride, calcium and magnesium (89–91). In a state of stress, there are higher concentrations of calcium in the extracellular fluids that lead to a greater contractility of skeletal and heart muscles (92). Moreover, Grigor et al. (93) found significantly higher plasma sodium concentrations after 5.25 h of transport in fed calves (136 mmol/l) compared with the unfed controls (133 mmol/l), but it is unclear what this relatively small difference in plasma sodium means.

Changes in sodium values might be useful as indicators of calf diarrhea. Calves with diarrhea generally have significantly lower concentrations of serum sodium than healthy controls (94). This is in accordance with Maach et al. (95), who found that calves with acute diarrhea had lower plasma concentrations of sodium and chlorine (131.2 ± 6.8 mmol/l, 95.6 ± 6.9 mmol/l, respectively) in their serum compared with healthy calves (140.0 ± 9.9 mmol/l, 103.3 ± 6.9 mmol/l, respectively). Calves with diarrhea can also have hyperkalemia as a consequence of the dysfunction of the Na+/K+ ATPase (96).

Seifi et al. (72) found that serum potassium concentrations >5.63 mEq/l in calves with diarrhea were associated with 4-fold increase risk of mortality. Moreover, hyper-kalemia (*K* > 5.8 mmol/l) can cause severe dehydration and can be associated with changes in body temperature homeostasis (97). As a consequence of the alteration of physiological mechanisms, calves might be more predisposed to develop severe diseases.

### Osmolality

Transported calves may also experience changes in their plasma osmolality, which is an indicator of the osmotic pressure of the plasma (30, 31, 34, 98). Knowles et al. (30) observed the highest values of plasma osmolality in young calves transported for 24 h (278 mOsm/kg) compared with control calves (275.4 mOsm/kg). These results indicated that calves become more dehydrated when they are subjected to a longer transport duration. The effect is likely to be more pronounced when calves are deprived of food and water prior to transport (30). Osmolality is, therefore, an important indicator of the hydration status of the animal (31, 99).

The dehydration status of a calf might be related with an increased likelihood to develop diseases at the veal farm (27). Griffin et al. (100) assigned calves suffering from dehydration, combined with malnourishment and exhaustion to a high-risk class, with a greater probability of becoming sick. Moreover, Renaud et al. (28) reported that the degree of dehydration at arrival is an important predictor of mortality in the first 21 days after arrival at the veal farm.

### Hemoglobin

The white veal calf industry has always raised calves with a low hemoglobin (Hb) status. Hb acts as a transporter of oxygen from the lungs to the tissues and as a transporter of carbon dioxide from the tissues back to the lungs. This function depends on the molecular structure of hemoglobin, which contains four heme groups, each with a central iron molecule (101). Due to the specific diet based on milk replacers and solid feeds with low iron content for several weeks, calves might develop anemia, which has a negative impact on growth and feed conversion ratio (102, 103). However, in the interim period or at the end of the fattening cycle, lower Hb concentrations are desirable because they are associated with low myoglobin and pale meat (104).

Nowadays, the industry pays more attention in maintaining Hb values a certain range. In the Netherlands, all calves are monitored within the first 2 weeks upon arrival at the veal farm and calves with Hb levels below a certain threshold are treated with supplemental iron. Another systematic monitoring is done between 12 and 14 weeks of fattening (105). However, outside these moments blood Hb (or iron) values of veal calves are usually not systematically monitored, and it is likely that some animals may develop subclinical anemia. Therefore, measurement of Hb can be used to assess on-farm anemic state of calves.

Hb might also be used as predictor of diseases in calves. As a consequence of their anemic status, both health and robustness of calves are affected and calves are more vulnerable to diseases (106). In animals iron deficiency (with blood Hb lower than 6.0 g/dl) is known to affect both humoral and cell-mediated immunity (107). As a consequence of the impaired immune functionality, calves might be more susceptible to infectious diseases. Steinhardt and Thielscher (108) observed that the growth of calves with low Hb values at arrival at the veal farm was lower compared with calves with normal range of Hb. Gygax et al. (103) reported also that iron deficiency can lead to higher infection rates, especially in the respiratory and the gastrointestinal tract. Therefore, these findings suggested that Hb values upon arrival at the veal farm may be good biomarkers for predicting calf health and welfare status after transportation to the veal farm; lower Hb levels are indeed more likely to be associated with a major risk of poor welfare and a higher incidence of diseases (109, 110). Hence, the deliberate attempt by the veal industry to avoid anemia in young calves is through the administration of iron.

### Conclusions on dehydration-related variables

All variables discussed in this paragraph are correlated with dehydration and PCV seems to be the most suitable and practical on farm biomarker. However, their association with disease status may not only be based on dehydration status, but also on factors like stress, colostrum intake, acute phase proteins and antioxidative status. Therefore, more research on correlations among these variables and the occurrence of respiratory or enteric diseases would be necessary to perform. Based on reasearch currently reviewed, it can be disputed which dehydration related variable is most reliable as on-farm biomarker.

## Energy and protein metabolism

### Lactate

Besides dehydration, losses of glycogen, protein and fat may affect metabolism and future diseases in calves. In order to understand the consequences of body glycogen, protein and fat mobilization on future health problems, different variables can be measured. Calves may experience a rise in lactate levels, especially after a long-distance transport (111). This might be the result of the degradation of muscle glycogen due to stress or exhaustion, that causes the liberation of catecholamines and a rapid glycogenolysis and gluconeogenesis (112).

In a study in cattle (49), L-lactate levels were higher prior to viral infection. Moreover, higher L-lactate values are related with higher risk of disease occurrence, like displaced abomasum and volvulus, in different animal species (113–115), and especially in calves with diarrhea (116–118). A positive correlation (*r* = 0.55) between acidosis (determined by L-lactate) and dehydration (determined by PCV) was found in calves with diarrhea (119).

**Figure 3:**
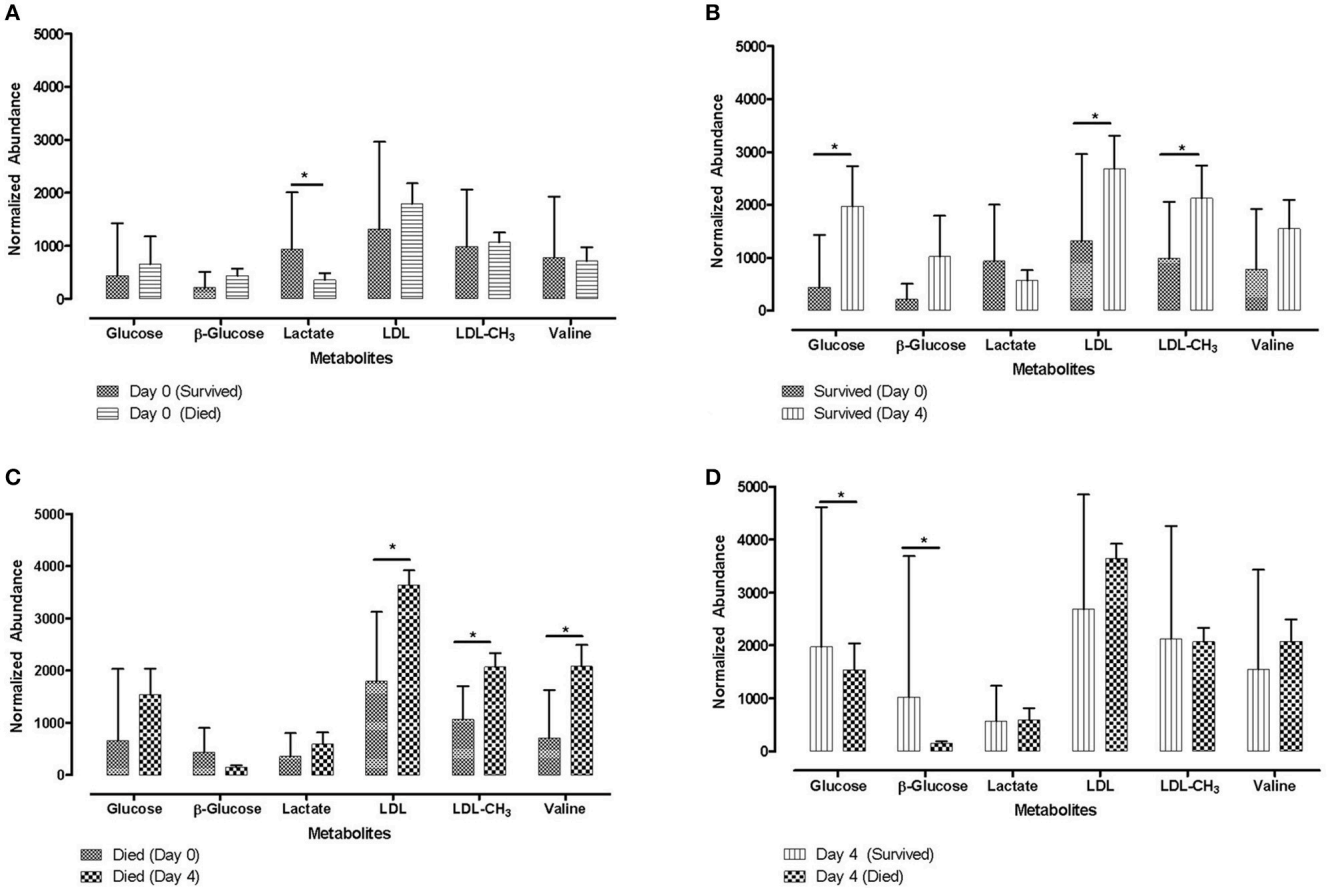
Metabolite profiles for animals that survived or died. Letters **(A)** to **(D)** represent four different situations depending on metabolite data derived from samples collected on Day 0 (prior to viral infection) and samples collected on Day 4 (post BHV-1 infection). Bar charts for distribution profile of identified metabolites from ^1^H-NMR studies for animals that died or survived following synergic viral-bacterial infection are shown in bar chart form. Error bars shown indicate 1-standard deviation. Metabolite IDs are shown on the x-axis [The publisher for this copyrighted material is Mary Ann Liebert, Inc., publishers Aich et al. (49)]. ^*^Significant differences *p* < 0.05.

Associations between lactate concentrations and measures of later calf health were also reported by different studies, with contradictory results. Aich et al. (49) found that calves which survived had significantly higher lactate concentrations prior to a viral infection compared to calves which died (Figure 3A). In contrast, in a study by Coghe et al. (120), relatively high lactate levels (>4 mmol/l) in BRD affected calves were associated with an increased likelhood of mortality in the following 24 h.

Buczinski et al. (121) reported that for each 1-unit increase of the log-lactemia there was an increase of 36.5 in hazard of dying of BRD. In general, enhanced L-lactate levels are associated with hypoxemia (122) and /or endotoxemia (123) that characterizes ongoing BRD episodes. Lower oxygen levels in the lungs are known to reduce macrophage activity, so pathogens can multiply at higher rates. As a result, the animal might be more exposed to the action of pathogens and it might develop respiratory diseases (124).

Thus, L-lactate might be considered a biomarker for assessing an ongoing disease process, such as pneumonia (based on clinical signs and hypoxemia) and for predicting death of clinically ill calves within 24 h. However, contradictory results merit further research into the use of L-lactate and lactate as biomarkers for respiratory diseases in young veal calves. At the same time, practical application of this biomarker might be highly feasible, because it can be measured by a portable analyser at a relatively low cost and results are available in 60 s (121).

### β-Hydroxybutyrate (β-HB) and non-esterified fatty acids (NEFA)

Environmental challenges, as depicted in Figure 1, are the main cause of mobilization of fat resources in young calves (125). The mobilization of the adipose tissue is associated with an increase in plasma concentration of free fatty acids and β-hydroxybutyrate (β-HB) (31, 62). Transportation as well as fasting increase the energy demands of young veal calves, so animals experience hypoglycaemia and a lack of C3 units in mitochondria (126, 127). As a result, C2 units accumulate in the mitochondria and are removed via production of ketone bodies. Bernardini et al. (48) found an increase in NEFA and β-HB above the normal values (0.13–0.20 mmol) at the end of transportation. These results were in accordance to Radostits et al. (128) and Knowles et al. (30), who found concentrations up to 0.46 mmol/l β-HB and 0.55 mmol/l NEFA in young calves (between 1 and 2 weeks of age) transported for 24 h. Moreover, the increase in NEFA and β-HB appears to be greater in unfed calves compared with calves that receive feeding prior to transport (30).

β-hydroxybutyrate and NEFA values represent useful indicators of calf energy balance and of body fat mobilization during and immediately after transportation of calves to the veal farm (34).

Changes in energy balance of calves might be associated with changes in biochemical, endocrinological and metabolic pathways underpinning production, maintenance of health and ability to cope with disease challenges (129). According to Wilson et al. (76) and Renaud et al. (28) many male calves entering the veal facility are experiencing suboptimal energy status and low body fat cover. A suboptimal energy status and higher levels of NEFA in the serum facilitate disease development and suppress the immune function (130). Future studies should continue to investigate the complex link between the immune system responses associated with increase in blood NEFA levels and β-HB concentrations. So far, associations between negative energy balance, NEFA and β-HB have only been studied in adult dairy cattle (131), and there is little information available on the effects of these parameters on disease incidence later in life of young veal calves. Renaud et al. (28) reported that NEFA and β-HB might be used as markers of the energy status, but in this study they were not associated with morbidity or mortality. However, this study was based on short transport duration, thus more research is needed to explore the effects of long transport duration on energy status.

### Urea

Plasma urea concentrations might also be affected by environmental challenges (Figure 1), including transportation (36). Higher plasma urea values are indicators of protein and nucleic acids breakdown in the muscles as a result of increasing cortisol concentrations and prolonged fasting (29). Knowles et al. (30) observed that young calves (<1 month old) receiving 1 l of glucose/electrolyte solution during transport showed lower urea (3.58 g/l) values after 24 h transport compared with the control group (4.50 g/l). In another experiment, these authors found higher plasma urea values in calves subjected to the same transport duration (5.61 g/l in 24 h) when not being fed during the journey compared with control calves (5.34 g/l) (30). These results suggest that feeding during long transport durations may help calves in reducing muscle protein degradation during their journey to the veal farm.

Urea concentrations might be used to assess the acute disease state of calves. In fact, protein catabolism, growth retardation and excessive nitrogen excretion might be the consequences of an ongoing disease process (132). However, additional information is needed to understand the underlying physiological and immune mechanisms involved in disease incidence.

Fayet and Overwater (133) analyzed several biochemical parameters of newborn calves and associated these variables with future survival of calves. Among these variables, blood urea concentration was a reliable predictor of survival rate of calves with 80% accuracy. The authors found significant differences in average urea concentrations between surviving calves (68.5 ± 35.7 mg/dl) and dead calves (141.4 ± 78.0 mg/dl). Seifi et al. (72) conducted another study in young calves (up 14 days of age) with diarrhea. The authors aimed to investigate the associations between serum biochemical variables and future survival of calves. The results of this study showed that blood urea nitrogen (BUN) concentrations can be used as valid prognostic indicators. Calves with diarrhea were 5.6 times more likely to die when their BUN concentrations were higher than 13.07 mmol/l. Correspondingly, Klee et al. (73) also demonstrated that the efficacy of treatment in calves with diarrhea was lower when BUN concentrations were above 28.56 mmol/l. However, given these differences in threshold levels, additional research is necessary before urea could be used in practice as biomarker.

### Glucose

As indicated in Figure 1, higher cortisol and glucocorticoids concentrations following transportation or other challenges might cause changes in plasma glucose. Previous studies on changes of plasma glucose concentrations in transported calves revealed different outcomes. At one hand, some authors (74, 134–136) found higher plasma glucose levels which may be a result of elevated stress levels that activate the hypothalamic-pituitary-adrenal axis (HPA). On the other hand, Trunkfield and Broom (137) showed that calves experienced hypoglycaemia after transportation, which may be a consequence of the activation of HPA axis, higher glucocorticoids and catecholamine concentrations and because of the higher energy requirements during the journey and restriction of food and water intake before, during and after transport (65). Mormede et al. (33) reported a decrease of 38 and 54%, respectively in plasma glucose level in young calves after short and long transportation. Alteration in plasma glucose concentrations are highly influenced by the time and plane of feeding before and after transportation of calves (29). Differences in this respect may explain the great variability among studies on glucose in calves after transport. On a short term and shortly after feeding, higher cortisol concentrations can cause an increase in plasma glucose, whereas on a longer term, glycogen stores can be depleted and thus blood glucose concentration is lower.

Studies in cattle reported that a disease challenge initially resulted in hyperglycemia, followed by a period of hypoglycemia (138, 139). Montgomery et al. (140) found lower plasma glucose levels than normal (5.3 ± 0.07 mmol) in an experiment with heifers treated for BRD. Moreover, hypoglycemia was found to be related to neonatal calf diarrhea and endotoxaemia in calves (141). However, more research should be done to investigate the association between hypoglycemia and acute diarrhea in veal calves.

Cusack et al. (142) found that low plasma glucose levels in calves on arrival at the feedlot were associated with a greater probability of developing severe BRD at later stages. This is in accordance with another study on steers transported for 12 h, in which low blood glucose levels after transport increased the incidence of morbidity and mortality in these animals in the following 56 days (71). Additionally, in the study of Trefz et al. (141) calves with severe hypoglycemia had a lower survival rate (20.6%) compared with calves with normal plasma glucose concentrations (74.0%). Mormede et al. (33) also reported that hypoglycaemia after transport in combination with lower growth rate in the first weeks after transport may even affect performance at later stages. Accordingly, Aich et al. (49) demonstrated with metabolomic analyses that higher glucose concentrations in calves of 6 months of age, following a viral BRD infection predicted survival of the animals. Against this background, several studies showed the potential of glucose as important predictor. Therefore, lower glucose concentrations might be used as on-farm biomarker of future diseases in veal calves.

### Body temperature

Temperature homeostasis is important in order to guarantee the functionality of the main physiological mechanisms in the animal body. Young calves have a limited ability to regulate their body temperature, especially during transportation (143, 144). According to Hemsworth et al. (144), the thermal comfort zone of young calves is between 13 and 26°C, and they are sensitive to both heat and cold stress (145). Elmer and Reinhold (146) observed that young calves up to 6 weeks of age are the least tolerant to high ambient temperature (35°C) compared to older animals, especially during long journeys. Changes in rectal temperature can be caused by acute or chronic secretion of catecholamines and glucocorticoids (147). In case of acute stressors, such as handling and loading procedures prior to transport, rectal temperature can increase as a result of peak production in glucocorticoids and cathecolamines (147), whereas, prolonged transport, can decrease rectal temperature to normal temperature (148).

Garcia et al. (149) classified calves with pneumonia based on rectal temperature ≥39.5°C accompanied by clinical signs of respiratory disease (e.g., mucopurulent nasal discharge, cough, increased respiratory rate). McGuirk and Ruegg (150) also reported that rectal temperature higher than 39.4°C for two successive days in combination with a slower, lower or lack of milk intake are indicators of diseases. Particularly, rectal temperatures >41°C are associated with pneumonia (150). However, as reported by Galyean et al. (151), rectal temperature might be influenced by other factors as well, including processing order, crowding, ambient temperature and humidity. These factors should be considered when assessing animal performance and, especially BRD incidence, on the basis of rectal temperature.

Grigor et al. (31) found a positive correlation (r_s_ = 0.649, *P* < 0.01) between rectal temperature of young calves during the first week after transport and first week post-transport clinical respiratory disease score (points from 0 to 3, and greater scores were associated with a worse health status). Higher rectal temperatures in transported calves seemed to indicate a greater clinical response to either BHV-1 inoculations or to other infections. Calves might also experience hypothermia, which might be considered as an adaptive response related to post-transport severe dehydration and hypoglycemia. Hypothermia might cause impairment of the Na+/K+-ATPase, which is temperature dependent, and as a result animals might be more susceptible to diarrhea or other health problems (152).

Collectively, these findings show simple associations between rectal temperature and current health status of calves but not a clear predictive value of rectal temperature. However, both high and low rectal temperature might be associated with future health problems; future studies could investigate whether the negative effects are more pronounced in relation to high or low rectal temperature. With regards to feasibility, measurement of rectal temperature is a very easy, quick and non-invasive approach, thus it can be used by farmers on a frequent basis to check the health state of calves.

## Immunity

### Leukocyte count and other immune responses

As shown in Figure 1, environmental challenges affect circulating glucocorticoids (increase in cortisol levels) in calves. As a consequence of stress-driven higher concentrations of stress hormones, the immune system might be affected. On the one hand, acute stress might result in immuno-preparatory conditions, by helping the animal to reinforce its defense against pathogens; on the other hand, long-term exposure to stress might have immunosuppressive effects by making calves less resilient to diseases (153). Therefore, in case of chronic stress, the functionality of the immune system might be impaired and calves might have changes in number of leukocytes, neutrophils in the peripheral circulation and other immune cells (46, 154). An increase in neutrophil and mononuclear cell ratio (N:M) was reported (155, 156). An increase in number of neutrophils was also reported (153, 154), but results vary among studies and other authors showed the contrary (48). Moreover, white blood cells (WBC) decreased after transportation (112).

These changes in different immune parameters in the long-term might indicate an immuno-suppressive effect and they might affect the adaptive capacity of the animal to the environmental circumstances. Due to a low functionality of defense immune mechanisms, the animal might have a reduction in performance, weight gain and an increase in susceptibility to diseases (especially respiratory diseases, such as BRD) (154, 157, 158). Lower immunoglobulin (Ig) concentrations, especially IgG, of calves upon arrival at the farm was also reported and may contribute to a decreased resilience of calves to diseases (33). Pardon et al. (79) reported that concentrations of immunoglobulins (Ig) upon arrival at the farm, which are dependent on colostrum intake, may serve to predict BRD hazard in veal calves. Calves with Ig < 7.5 g/l have a greater probability of dying in the first weeks at the farm. However, no relationships were found between Ig concentrations and neonatal calf diarrhea. Interestingly, Renaud et al. (28) recently demonstrated that, similar to IgG, also greater concentrations of cholesterol were associated with lower risk for mortality in the first 21 days at the veal farm. Although this association could have multiple explanations, it was argued that cholesterol could be used as a marker of colostrum intake.

Overall, it could be concluded that measures of immunocompetence may be important predictors of later life performance, health and welfare. However, it is unclear which immunological variable or set of variables on-arrival at the veal farm would be the best predictor and thus suitable as biomarker.

### Acute phase proteins (APPs)

Acute phase proteins (APPs) are proteins synthesized in the liver. The release of APPs in the bloodstream is induced by cytokines in response to many stressors, including transportation (80). Cytokines are produced by cells of the innate immune system (e.g., macrophages, monocytes) and function as messengers between the local site of injury and the hepatocytes producing APPs (85, 159). APPs exert defensive roles against pathological damage, they are responsible of restoring the homeostasis and they regulate different stages of inflammation (80). Therefore, APPs can be considered as biomarkers of stress and immunity (154).

An increase in APPs is observed in animals with diseases, thus high levels of APPs might be used as quantitative measure for identifying sick calves (160). In calves with diseases, the main changes in circulating APPs involve serum amyloid A (SAA), haptoglobin (Hp), lipopolysaccharide binding protein (LPB), fibrinogen (Fb), α-1-acid glycoprotein (AGP) and lactoferrin (154). Gånheim et al. (161) indicated a threshold to distinguish between healthy and calves with diseases (age 9–18 weeks), including 0.13 g/l for Hp, 25.6 mg/l for SAA and 6.45 g/l for fibrinogen. Calves with a clinical or subclinical infection showed prolonged higher APPs concentrations than healthy calves (154). Godson et al. (162) showed that higher concentrations of Hp were associated with severe bacterial respiratory infections in cattle (*r*^2^ = 0.481). Angen et al. (163) and Hajimohammadi et al. (164) used Hp to identify calves with pneumonia and diarrea, respectively. Moreover, Tothova et al. (165) reported that the increase in some APPs levels (Hp and SAA) was associated not only with acute diseases of the respiratory tract, but also chronic cases (*P* < 0.01). The magnitude and the duration of the acute phase response reflect the severity of the infection (166–168).

Changes in APPs levels can be used not only as a measurement for early diagnosis and prognosis or for assessing the severity of diseases but also as predictors (81–83). Different studies suggested that Hp might be the most useful APP for predicting diseases and for discriminating between diseased and healthy calves due to the higher sensitivity in detecting diseases (163, 169, 170). Higher levels of Hp (more than 0.13 g/l) in the first week of life were reported to be related with an increased odds ratio of future treatment for BRD [odds ratio (OR) = 2.66; *P* = 0.048], treatment for other diseases (*OR* = 12.59; *P* < 0.001) and death (*OR* = 8.67; *P* = 0.001) (160). In contrast, SAA might be a less suitable predictor of health problems due to its higher sensitivity to stimuli other than diseases, such as stress (81, 171, 172). Therefore, higher values of acute phase proteins, especially Hp, might act either as indicators of an ongoing disease process or as predictors of diseases, thus they might be suitable as biomarkers.

## Enzymes

Along with changes in immune functionality, transportation of calves at the veal farm and handling procedures are the main cause of other changes in blood plasma, including higher levels of creatine kinase (CK), creatine phosphate kinase (CPK), lactate dehydrogenase (LDH) and aspartate aminotransferase (AST) (36). These enzymes play a significant function in energy homeostasis of tissue cells and they ensure a constant ATP levels in the cells (173). Changes in enzyme concentrations may occur due to physical and psychological challenges during transportation, which disrupt the homeostasis and, as a consequence, the metabolism of calves (174–176). In particular, higher plasma concentrations of CK, CPK and AST are associated with tissue damage, poor muscular tissue reperfusion, hypoxia and fatigue, and an increased permeability of muscles membrane following handling procedures and transport stress (65, 176–178).

Changes in plasma enzymes concentrations might function as indicators of tissue damage in diseased animals (179). However, information on the relationships between these enzymes and future diseases is lacking in calves and cattle. In human patients, CK concentrations are useful for the evaluations of disorders involving damage to the myocardium, skeletal muscle and central nervous system (180). Moreover, evaluation of blood values of this enzyme might be useful to discriminate between high-risk new-born infants and low-risk new-born (180). In other studies in humans, an increase in blood LDH values seemed to be an important indicator of lung damage, pulmonary endothelial cell injury or airways problems (181, 182). Additionally, AST is strongly related with hepatic function, thus higher AST concentrations may indicate liver problems (183). Values of AST are also assessed together with CK values for diagnosis of muscle damage (99). An increase in CK, LDH and AST on arrival of calves at the veal farm was also reported compared with pre-transport values (32, 33, 184). It can be speculated that, as in humans, higher values of these enzymes in veal calves might be associated with health problems, diseases and muscle damage. However, higher levels of these enzymes could also be positive indicators of restored homeostasis after transport or other challenges. In fact, when calves are stressed, they might try to restore their homeostasis by changing several physiological processes, including the enhancement of CK, LDH, and AST.

## Conclusions and future perspectives

Among all variables listed in this review, there are some that seem to be good predictors of future diseases because there is information available from published studies. These variables include PCV, BW, lactate, glucose, Ig and Hp. Other variables, including Na, osmolality, neutrophils and enzymes seem to be just indicators of ongoing diseases but they do not show any concrete association with future diseases. Existing literature suggests that variables such as cortisol, albumin, bilirubin, K, Hb, BW loss, β-HB, NEFA, urea and rectal temperature may act as potential biomarkers. However, due to limited data, more studies are needed to confirm their association with future diseases. Furthermore, as already indicated, different parameters may be correlated. In the study of Turkson and Ganyo (70), Hb was positively correlated with PCV vales (*R*^2^ = 0.5504). Moreover, the authors suggested to use the simplified relationship of Hb (g/dl) = (0.3 PCV) + 3 to estimate Hb concentration from PCV in cattle. PCV measurement is a simple and cheap approach and therefore it can be used by farmers to assess anemic status and dehydration of calves at the veal farm. Blood collection for Hb analyses is performed on routine basis in the veal industry. Different blood samples are collected at specific time points throughout the rearing period. We suggest that these blood samples may also be used to obtain potential biomarkers, including PCV.

With regards to Hp, concentrations of this *APP* > 0.13 g/l in combination with higher rectal temperature, increased nasal score and calf depression are indicators of respiratory diseases such as pneumonia (160). Overall, generation of big data sets including all the afore mentioned variables would allow to establish correlations between all different parameters. With these datasets, it would be possible in future studies not only to describe associations between variables, but also to find their predictive value for diseases in later life in veal calves. Eventually, the performance of promising biomarkers of health and performance of veal calves should also be investigated in terms of quantitative test characteristics such as sensitivity and specificity.

There are other new alternatives that could be considered to obtain more information on these variables and thus improve on farm health problems. A solution might be the use of post-genomic technologies of transcriptomics, proteomics and metabolomics in order to develop new biomarkers for detecting diseases (185, 186). So far, only a limited number of post-genomic strategies were applied in veterinary research (187), but their use is of growing interest in animal studies (188). Transcriptomics might be used for detecting and genotyping animal pathogens and for studying gene mutation in case of diseases (188, 189). Within this field, DNA microarrays and analysis of gene expression are interesting tools for the identification of potential biomarkers of future diseases (185, 188). Proteomics might be a useful approach in biomarker discovery by studying protein component of a cell, tissue and organism (188, 190). Several techniques have been adopted in proteomic studies and quantitative proteomic strategies are developing with the aim to be applied in biomarker research. For instance, Aich et al. (49) analyzed the associations between apolipoprotein AI and haptoglobin with the risk of developing respiratory diseases in cattle. Metabolomic studies focus on low molecular weight metabolites and might be used to investigate pathophysiological processes of animal diseases (191, 192). Aich et al. (49) used, for example, lactate and glucose analyses to predict BRD outcome in cattle. Overall, post-genomic technologies are becoming more accepted in veterinary studies, but due to many problems related to their feasibility, cost and practicality the use of these technologies in practice is still limited. Therefore, further research in these fields is needed to identify biomarkers by using more practical and feasible solutions that can be applied in routine clinical practice.

## Author contributions

FM wrote the review (main body) and HvdB, BK, and KvR contributed during the discussion and reviewed the preliminary versions of the manuscript.

### Conflict of interest statement

The authors declare that the research was conducted in the absence of any commercial or financial relationships that could be construed as a potential conflict of interest.
